# Novel method to confirm tracheal intubation based on airway pressures: validation in patients with lung disease

**DOI:** 10.1186/cc12088

**Published:** 2013-03-19

**Authors:** A Stemerdink, KG Monsieurs, F Luijmes, W Dieperink, JG Zijlstra, AF Kalmar

**Affiliations:** 1 University Medical Center Groningen, the Netherlands; 2 Antwerp University Hospital, Antwerp, Belgium

## Introduction

Emergency endotracheal intubation results in accidental oesophageal intubation in up to 17% of patients often with disastrous consequences. We have previously published a highly specific and sensitive novel method to detect endotracheal intubation based on differences in ventilation pressure waveforms in the oesophagus and in the trachea in patients with healthy lungs [1]. A detection algorithm, based on differences in compliance/elasticity between the lung and the oesophagus, generated a D-value indicating tracheal intubation if D >0.5 and oesophageal intubation if D <0.5. The aim of the current study was to validate the algorithm in patients with lung disease.

## Methods

After obtaining institutional approval, 20 intubated and ventilated ICU patients were included. Inclusion criteria were controlled mechanical ventilation and at least mild to moderate lung injury according to a Murray lung injury score >0.1. A connecting piece was placed between the endotracheal tube and the ventilation bag. This piece comprised a thin air-filled catheter inserted through the tube lumen at 1 cm from the distal end, and a second catheter located at the proximal end of the tube. We performed three consecutive manual bag ventilations while recording the pressure curves through both catheters. For each ventilation, a D-value was calculated.

## Results

**Figure 1:**
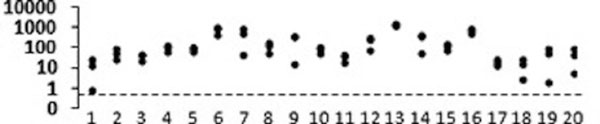
D-value of individual test ventilations.

Mean age (SD) of the patients was 60 (16) years, 60% were male. The mean (SD) Murray score was 1.4 (0.6). Pathologies included pulmonary oedema, pneumonia, atelectasis and traumatic lung injury. All 60 D-values are represented in Figure 1. The median (IQR, range) D-value was 38 (16 to 74, 0.8 to 1,272). Our algorithm therefore confirmed a high sensitivity to detect correct endotracheal intubation also in patients with lung disease. Under the hypothesis that oesophageal compliance does not increase significantly in patients with lung disease, the specificity of our algorithm will not be affected.

## Conclusion

The algorithm to detect correct endotracheal intubation performed excellent in patients with lung disease.
